# *Pearsonema* spp. (Family Capillariidae, Order Enoplida) Infection in Domestic Carnivores in Central–Northern Italy and in a Red Fox Population from Central Italy

**DOI:** 10.3390/ani10091607

**Published:** 2020-09-09

**Authors:** Salvatore Pelligra, Lisa Guardone, Francesca Riggio, Francesca Parisi, Michela Maestrini, Alessia Mariacher, Stefania Perrucci

**Affiliations:** 1 Dipartimento di Scienze Veterinarie, University of Pisa, 56124 Pisa, Italy; s_pelligra@hotmail.it (S.P.); lisa.guardone@for.unipi.it (L.G.); frapam83@tiscali.it (F.R.); francesca.parisi@vet.unipi.it (F.P.); michela.maestrini@phd.unipi.it (M.M.); 2 Istituto Zooprofilattico Sperimentale delle Regioni Lazio e Toscana, Centro di Referenza Nazionale per la Medicina Forense Veterinaria, 58100 Grosseto, Italy

**Keywords:** *Pearsonema plica*, *Pearsonema* spp., dog, cat, red fox, Italy

## Abstract

**Simple Summary:**

*Pearsonema* spp. nematodes live in the urinary bladder and sometimes in the ureters and renal pelvis of domestic and wild carnivores, in which they may be responsible for urinary capillariosis. While the red fox is considered a reservoir of *Pearsonema plica*, the occurrence of *Pearsonema* spp. infection in domestic carnivores is still poorly known. This study aimed to assess the occurrence of *Pearsonema* spp. infection in privately owned cats and dogs from central (Tuscany) and northern Italy (Piedmont and Lombardy) alongside its occurrence in a red fox population from central Italy (Tuscany). Among the examined animals, 2/26 cats (7.7%), 1/83 dogs (1.2%), and 38/42 foxes (90.5%) were found positive. Recurrent cystitis, pollakiuria, and hematuria were the main clinical signs in symptomatic dogs and cats. This is the first study on *Pearsonema* spp. infection of domestic carnivores examining a large number of privately owned pet animals. Obtained results confirm the role of the red fox as a reservoir for *P. plica* in Europe and suggest a possible high occurrence of *Pearsonema* spp. infection in domestic cats of central Italy.

**Abstract:**

*Pearsonema* spp. nematodes infect the urinary bladder of domestic and wild carnivores. The red fox is considered a reservoir of *Pearsonema plica*, while the prevalence of *Pearsonema* spp. in domestic carnivores is still poorly known. This study aimed to assess the occurrence of *Pearsonema* spp. infection in privately owned cats (26) and dogs (83) from central and northern Italy alongside occurrence in red foxes (42) from central Italy. In positive dogs and cats, associated clinical signs were also evaluated. Urine samples were first examined under a stereomicroscope; then, they were centrifuged and microscopically examined after a flotation test. As for foxes, the urinary bladders were opened and urine was collected and processed as above, while collected nematodes were identified at the species level. Among examined animals, 2/26 cats (7.7%), 1/83 dogs (1.2%), and 38/42 foxes (90.5%) scored positive. Recurrent cystitis, pollakiuria, and hematuria were the main clinical signs in symptomatic dogs and cats. This is the first study on *Pearsonema* spp. infection in domestic carnivores examining a large number of privately owned pet animals. Obtained results confirm the role of the red fox as a reservoir for *P. plica* and suggest a possible high occurrence of *Pearsonema* spp. infection in domestic cats of central Italy.

## 1. Introduction

According to the more recent and worldwide accepted taxonomic revision of the Family Capillariidae, nematodes affecting the urinary tract of domestic and wild canids and felids only include two species: *Pearsonema plica* and *Pearsonema feliscati* [1,2]. However, a third urinary capillariid species, namely *Capillaria travassoi*, has been reported in domestic felids in North America, but it is still not clear whether *P. feliscati* and *C. travassoi* should be considered distinct species or the same species [3]. Adults of these capillariids reside in the urinary bladder and, more rarely, in the ureters and renal pelvis of domestic and wild carnivores, in which they may be responsible for urinary capillariosis [4,5,6,7,8]. The host spectrum of *P. plica* includes wild and domestic canids and felids, while *P. feliscati* only infects felids [3,9,10]. The life cycle of *P. plica* is indirect and includes earthworms as intermediate hosts, in which the eggs shed by the final host with the urine develop into larvae that are able to infect the carnivore-definitive host [3,9,10,11,12]. Putative paratenic hosts are believed to be able to amplify the transmission of the infection [2,3,11,12]. The life cycle of *P. feliscati* is still unknown, but it is thought to be similar to *P. plica*, while no information is available about the life cycle of *C. travassoi* [3,12].

*P. plica* infections in Europe are frequently observed in the red fox (*Vulpes vulpes*) and less commonly in the wolf (*Canis lupus*), brown bear (*Ursus arctos*), golden jackal (*Canis aureus*), and some mustelid species [4,5,6,7,13,14,15]. More specifically, high prevalence rates, up to 90% [4,5,6,15,16,17,18], are frequently observed in red foxes in Europe, and this wild carnivore is considered a potential reservoir for *P. plica* infection in dogs and cats [5,15]. In Italy, *P. plica* infection in the red fox has been known for a long time, with prevalence rates ranging from 0% to 56.8% in different areas of the Italian territory [6,16,17].

As for domestic carnivores in Europe, a high risk of infection is thought to occur in dogs and cats living in urban, peri-urban, and wooded areas frequented by red foxes [6,18]. However, few epidemiological studies have been conducted in domestic dogs [19] and cats [20,21,22,23,24], and worldwide reports of *Pearsonema* spp. infections in pet domestic carnivores mostly consist of scattered clinical cases [10,25,26,27,28,29,30,31], also from Italy [8,11,32,33].

*Pearsonema* spp. infection in domestic animals is usually diagnosed by microscopic analysis of the urinary sediment through the identification of *Pearsonema* spp. eggs based on their morphological characteristics [8,10]. Urinary samples can also be examined under a stereomicroscope prior to microscopic analysis, to detect mature or immature parasites and/or their fragments [11,32], but this analysis is not usually performed by veterinary clinicians. However, the diagnosis of the infection can be somehow problematic due to several factors: the long prepatent period; the high occurrence of asymptomatic and subclinical infections caused by a low parasitic load, which is often associated with inconstant elimination of a low number of eggs; immature eggs not being easy to find or identify; the clinical signs often being similar to that caused by urinary bacterial infections; and, frequently, also, the unawareness of veterinary clinicians about *Pearsonema* spp. infections [2,8,10,11,19,32,33]. Hence, dog and cat *Pearsonema* spp. infections may be underestimated. Nevertheless, in dogs and cats, the infection can present with overt clinical signs, especially if complicated by the intervention of other factors, mainly concurrent bacterial infections [10,11,25,27,28,33,34]. Dysuria, pollakiuria, hematuria, pyuria, and urine incontinence are the most commonly observed clinical signs [8,10,25,32,33]. However, more severe clinical signs, such as obstruction of the urinary tract and chronic renal failure with glomerular amyloidosis associated with *P. plica* infection, have also been reported in a cat and two dogs in Italy, respectively [8,11,32].

In wild carnivores, the infection is diagnosed at necropsy through the detection and identification of *Pearsonema* spp. adults and eggs by the removal, opening, and microscopic examination of the urinary bladder and its content [4,6,35,36].

In view of the limited knowledge on the occurrence of *Pearsonema* spp. infections in domestic carnivores, this study aimed to assess the occurrence and related clinical signs of *Pearsonema* spp. infection in privately owned dogs and cats from different areas of central and northern Italy. Considering the putative role of the red fox as a potential reservoir for *P. plica* infection in dogs and cats in Europe, the frequent presence of red foxes in urban and peri-urban areas throughout Europe, and the limited recent data available on *P. plica* infection in the red fox in Italy, a secondary aim was also to assess the frequency of this parasitic infection in a free ranging population of red foxes in the province of Pisa (Tuscany, central Italy), an area of Italy in which data about red fox *P. plica* infection are lacking.

## 2. Materials and Methods

### 2.1. Domestic Carnivores

Between May 2015 and March 2017, 26 pet cats and 83 pet dogs and of both sexes and different age that were referred to several veterinary clinics for various reasons were examined to assess urinary *Pearsonema* spp. infections. Inclusion criteria were as follows: (1) age > 1 year; (2) possibility of access to the external environment; and (3) no anthelmintic treatments received in the two months prior to the study.

For each animal, age, sex, breed, area of origin, clinical signs, urinalysis results, and, when possible, patient follow-up were recorded (Table 1).

From each animal, a urine sample of 2.5–3.5 mL was collected and examined. Samples were collected after mid-stream free catch, catheterization or cystocentesis by the veterinary surgeon. Analysis of collected samples included visual macroscopic evaluation for color and eventual turbidity. Subsequently, each sample was placed in a Petri dish and observed under a stereomicroscope to assess the presence of adults or immature parasite stages, which were collected, examined under an optical microscope, and identified on the basis of their morphometric features [8,9,11,34,35]. Then, each urine sample was centrifuged for 5 min at 400× *g*, the supernatant was discarded to 1 mL, and the sample was examined for the presence of *Pearsonema* spp. eggs under an optical microscope after a flotation test with 20 mL of saturated NaCl solution (specific weight 1.2). Eggs were identified based on their morphological features [6,9].

Written informed consent was obtained from all the owners of pet dogs and cats enrolled in this study.

### 2.2. Red Foxes (Vulpes Vulpes)

Between May 2015 and March 2017, 11 adult female and 31 adult male foxes were examined. All examined foxes were culled in the Province of Pisa, central Italy (43° N, 10–11° E), stored at 4 °C, and necropsied within 24 h. From all 42 necropsied red foxes, the urinary bladder was carefully opened, and urine was collected and processed as for domestic animals. If urine was absent, the bladder was washed with saline and the lavage fluid was examined. Moreover, all opened urinary bladders were observed under a stereomicroscope for the presence and collection of capillariid nematodes, which were identified at the species level based on their morphological features under a light microscope [3].

### 2.3. Statistical Analysis

For statistical analysis, domestic animals were divided in two age classes: 1–7 years old and >7 years old. Statistical differences for positivity to *Pearsonema* spp. were analysed using a Chi-squared test, considering sex, age, class, and geographical origin as variables in dogs and cats, and only sex in foxes. Statistical significance was set at *p* < 0.05.

## 3. Results

### 3.1. Cats

Examined cats (*n* = 26) included 19 mixed bred and 7 purebred animals of 1.5–14 years of age. Twenty/26 cats showed clinical signs related to diseases of the lower urinary tract (Table 1). Among these, 2/26 cats (7.7%, 95% CI, 0–17.9%) were found positive for *Pearsonema* spp. infection. No statistical differences were observed concerning sex, age, class, and geographical origin.

**Figure 1:**
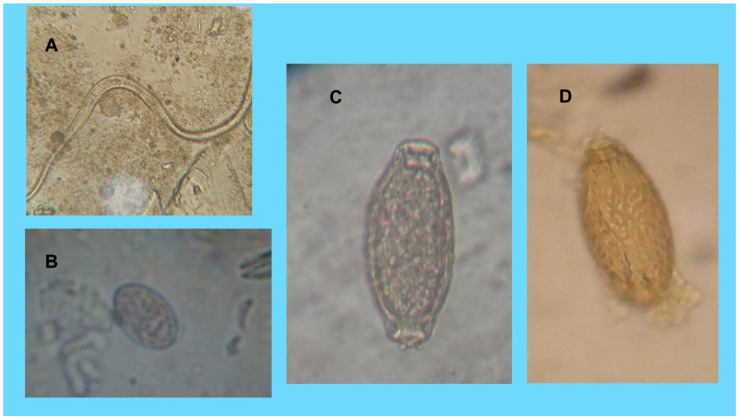
*Pearsonema* spp. infection in privately owned cats and dogs. (**A**) Nematode fragment detected in the cat living in Pisa (Tuscany) and identified as an adult of *Pearsonema* spp. for morphology and size. (**B**) Immature egg of *Pearsonema* spp. (31 µm long and 18 µm wide) identified in the urine sediment of the cat living in the province of Livorno (Tuscany). (**C**) and (**D**) Egg of *Pearsonema plica* in the positive dog from Milan (Lombardy): (**C**) Normal appearance of the egg (60 µm long and 25 µm wide) at microscopic observation; (**D**) Outer eggshell morphology.

The first positive cat was a 6 years old Persian neutered male cat from Pisa (Tuscany), which was referred for pollakiuria. The cat had a mainly indoor lifestyle but occasionally frequented an external communal garden. Clinical history revealed recurrent cystitis. No abnormalities were observed at physical examination. The urine was orange-yellow in color with turbidity, a specific gravity of 1.020, and a pH of 8.0. Proteinuria and hematuria were observed. At microscopic analysis of the urine sediment, a moderate level of leukocytes, a marked presence of erythrocytes, rare epithelial cells, a moderate presence of phosphate crystals, and cocci-type bacteria were observed. Moreover, in the urine sample, a nematode fragment was also detected and identified as a fragment of an immature mature adult of *Pearsonema* spp. for morphology and size (about 12 mm in length and 53 µm in width) (Figure 1A). A diagnosis was made of hemorrhagic cystitis by *Pearsonema* spp. with secondary bacterial infection. The cat was treated with antibiotics (Enrofloxacin 5 mg/Kg sid for 10 days), non-steroidal anti-inflammatory (Robenacoxib 1 mg/Kg sid for 6 days), and Fenbendazole (50 mg/Kg orally, once a day for seven days). Moreover, a food supplement (Urys^®^ Cur, Innovet Italia Srl, Milan, Italy) and a low-protein diet (Hill’s prescription diet c/d feline urinary stress) were prescribed. After treatment, clinical signs disappeared, urine parameters were in the normal range, and no *Pearsonema* nematode/eggs were detected at urine microscopic examination performed monthly in the following three months.

The second cat was an 8-year-old pet European male cat, living in the province of Livorno (Tuscany), an area bordering the area of the examined foxes. The cat was referred to a private veterinary clinic after a road traffic accident but was otherwise asymptomatic for urinary tract signs. The cat had a mainly outdoor lifestyle and was known to exhibit hunting behavior. At urine analysis, proteinuria, and hematuria, a specific gravity of 1.030 and a pH of 6.5 were evidenced. At microscopical examination, the urinary sediment showed a high level of leukocytes and erythrocytes. In addition, several nematode eggs measuring 31 × 18 µm on average, with an eggshell almost completely absent and two rudimentary poles, were also observed in the urine sediment (Figure 1B). For morphology and size, these eggs resembled *P. plica* immature eggs detected by Basso et al. [9] and Mariacher et al. [6] in infected dogs, and were identified as immature eggs of *Pearsonema* spp. This cat was treated with antibiotics (enrofloxacin 5 mg/kg sid and an association of amoxicillin and clavulanic acid 10 mg/kg bid), a non-steroidal anti-inflammatory drug (meloxicam 0.1 mg/Kg sid) and fenbendazole (50 mg/Kg orally, once a day for seven days). However, no follow-up information was available.

### 3.2. Dogs

Examined dogs (*n* = 83) included 28 mixed bred and 55 purebred animals of 1–16 years old, of which 62/83 dogs were showing clinical signs related to diseases of the lower urinary tract (Table 1).

Among examined animals, 1/83 dogs (1.2%, 95% CI 0–3.6%) was found positive for *Pearsonema* spp. infection. No statistical differences were observed about sex, age, class, and geographical origin.

The positive dog was a 2-year-old Pug male dog with indoor/outdoor lifestyle from Milan (Lombardy). The dog was referred for pollakiuria and had a history of recurrent cystitis. A urine sample was collected by catheterization, and it was intense yellow and semi turbid in appearance. Chemical examination revealed an increased urinary pH (8) and traces of proteins. Sediment microscopical examination revealed an increased presence of leukocytes and erythrocytes in association with struvite crystals, epithelial cells, and bacteria (cocci). Furthermore, *P. plica* eggs measuring 60 µm × 25 µm were found in the urine sediment (Figure 1C,D). The dog was treated with fenbendazole (50 mg/Kg orally, once a day for seven days), and after this treatment, clinical signs disappeared. *P. plica* eggs were not found at urine analysis performed a month later.

### 3.3. Red Foxes (Vulpes Vulpes)

Among examined foxes, 38/42 subjects (90.5%, 95% CI 81.6–99.4%), including all males (31) and 7/11 females, were found positive for *P. plica* adults and eggs. However, no statistical differences were observed regarding the sex.

**Figure 2:**
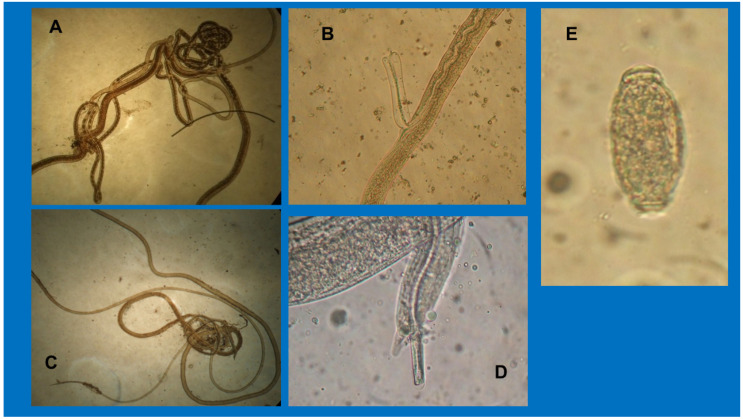
Adults and eggs of *Pearsonema plica* in red foxes. (**A**) Adult female of *P. plica* (43 mm × 62.4 µm); (**B**) Vulvar appendage (adult female); (**C**) Adult male of *P. plica* (27 mm × 50 µm); (**D**) Caudal ala and *spiculum* (adult male); (**E**) Mature egg (64 µm × 28 µm).

Since there are several difficulties in counting the exact number of worms, especially due to their fragmentation, we refrained from doing so. Adults were long, thin (Figure 2A,C), and their dimensions ranged within the length and width reported for *P. plica* [3,11]. Males of *P. plica* measured on average 27.7 mm in length and 50 µm in width, while the females measured 43 mm in length and 62.4 µm in width. More specifically, in the females, it was possible to observe eggs arranged in a pearl necklace and the external vulvar appendage, which on average measured 156 µm in length and 33.8 µm in width (Figure 2A,B). In the caudal end of the males, the spicules were about 2.2 mm long and 15.6 µm wide, and the digitiform papillae were surrounded by triangular caudal alae (Figure 2C,D). The observed eggs showed the typical morphological features of mature *P. plica* eggs, as they were lemon-shaped, colorless, with a shell with evident blocks and a thick texture and dimensions ranging from 60 to 68 µm in length and 24 to 30 µm in width (Figure 2E).

## 4. Discussion

In previous epidemiological studies evaluating *Pearsonema* spp. infection in domestic carnivores, only stray and shelter animals have been considered [19,20,21,23,24,37], while only reports of single or few animal clinical cases are available for dogs and cats kept as pets [6,9,10,23,24,25,26,27,28,29,30,31]. Therefore, this is the first study evaluating the occurrence of *Pearsonema* spp. infection in a larger number of domestic carnivores referred to veterinary clinics and including both symptomatic and asymptomatic animals. Obtained results showed the occurrence of *Pearsonema* spp. infection in 2/26 and in 1/83 examined cats and dogs, respectively.

The reported prevalence of *Pearsonema* spp. infection in stray or shelter cats in previous studies varies from about 6% (1/17) in Germany [19], to 67% (38/57) in Albania [18]. A *P. feliscati* prevalence of 18.3% (73/400) and of 6% (3/48) has been reported in Australia [22] and in the USA [36], respectively, while in Brazil, 5 out of 146 examined stray cats (3.4%) tested positive [21]. Thus, the occurrence of *Pearsonema* spp. found in cats in this study (7.7%) is higher than that previously reported in in stray or shelter cats in most studies worldwide [19,21,36]. However, it should be considered that most of the cats here examined (20/26) were symptomatic. On the other hand, it is also possible that in this study, some positive cats were not identified, because the excretion of eggs by adult *Pearsonema* spp. females can be intermittent and, especially with low parasite burden, more than a single urine sample should be analysed to improve the probability of diagnosis [38].

The only available epidemiological study on *P. plica* in domestic dogs is quite dated, and it concerns 127 dogs from two kennels where animals were frequently moved from one kennel to the other [17]. Therefore, the very high prevalence rates (76% and 59%) found in these two kennels examined in the USA were considered by the authors themselves as a peculiar epidemiological situation not representative of the actual occurrence of this parasite [19].

In Italy, reports of *Pearsonema* spp. in domestic carnivores refer to a few symptomatic clinical cases and concern an 8-month-old cat from central Italy [11] and eight adult dogs from different Italian areas [8,32,33]. Positive cases have also been reported in dogs and cats in other European countries, such as Holland [29,30], France [26], the United Kingdom [31] and, more recently, in Switzerland [10], Poland [36], and Slovakia [28]. The scattered case reports are possibly due to different reasons, such as asymptomatic cases or difficulties in diagnosis in live animals [2,8,10,11,19,32,33].

Frequently, *Pearsonema* spp. positive domestic carnivores have an outdoor lifestyle, as these animals are more likely to have contacts with intermediate and paratenic hosts in contaminated environments. Nonetheless, some recent studies have shown that the infection can also affect dogs that live mainly indoor [8,38]. As also hypothesized for other nematodes, mainly in dogs, this may be related also to the increasing tendency of owners to take their animals on vacations, often involving activities such as trekking and walking in the countryside, and possibly to the establishment of urban recreational environments closer to natural ecological systems [39,40].

In cats, *Pearsonema* spp. infection is frequently reported as responsible for pollakiuria, dysuria, and cystitis, sometimes hemorrhagic and frequently complicated by secondary bacterial infections [25,41,42]. Recurrent cystitis may also occur in cats affected by urinary capillariosis but left untreated or treated with ineffective drugs [11].

In this study, a fragment of an (immature or mature) adult of *Pearsonema* spp. for its length, width, and filamentous appearance, was detected at microscopical observation of the urine sample of a positive cat with a history of recurrent cystitis, and a hemorrhagic cystitis caused by *Pearsonema* spp. was diagnosed. The cat recovered from the infection after the treatment with fenbendazole, an anthelmintic drug that is considered effective for the treatment of urinary capillariosis [6,29,31]. To the best of our knowledge, this is the first report of *Pearsonema* spp. and, more in general, of nematodes in a cat urinary sample. On the contrary, the presence of nematode larvae in urine samples of dogs and humans has been previously reported, especially in cases of disseminated *Strongyloides stercoralis* infections [43,44], which is a nematode species that can affect also the cat [45]. The larger width of the nematode fragment found in the positive cat allowed its differentiation from *S. stercoralis* adults and larvae [12]. The absence of *Pearsonema* spp. eggs in the urinary sediment of this cat may depend on several factors, such as the presence of immature or “old” female parasites [8,19], and a consequent low number or an intermittent shedding of eggs with the urine. Although the cat of this case report was living in an urban environment, its habit of visiting the external environment may have enhanced the risk of infection.

Differently from the previous cat, the second positive cat was asymptomatic, and the detection of *Pearsonema* spp. eggs in the urine was an incidental finding. Indeed, in cats, this parasitic infection may frequently cause silent or subclinical forms that often remain undiagnosed [23,25,41,42]. Although the detection of immature *Pearsonema* spp. eggs has been reported in dogs infected by *P. plica* [8,10,38], this study is the first report in a cat. The morphological features of immature eggs can be very different from mature eggs, and their recognition is often difficult [6,9].

The only dog that was found positive in this study was a pet dog with a history of recurrent cystitis and living in Milan. As observed in other studies [8,10,28,30,33], in this dog, *P. plica* infection was associated with cystitis and pollakiuria. However, also in the case of dogs, the possibility that some positive cases have not been detected in this study should be considered, especially because only a single urine sample for each animal was examined.

Although no recent data about red fox populations in Italy is available, this wild carnivore species appears to be generally abundant on the Italian territory without any conservation problems, despite being regularly hunted and subjected to numerical control plans on a local scale [46]. Data obtained in this study showed a higher frequency of *P. plica* infection in adult red foxes of the province of Pisa (90.5%) than in red fox populations of other areas in Italy and Europe [4,5,6,15,16,17,47,48,49,50,51]. More specifically, in northern Italy, *P. plica* infection was observed in 0% (*n*. 93), 21.4% (*n*. 28), 27.6% (*n*. 29), 45.4% (*n*. 33), and 56.8% (*n*. 165) of examined red foxes respectively in Lombardy, Veneto, Trentino, Piedmont, and Liguria [6,16,17], while 14% (*n*. 182) of examined red foxes scored positive in Latium, central Italy [16].

The results from this study confirm the role of the red fox as a reservoir host for *P. plica* in Europe [4,5,6]. Moreover, considering the increased presence of red foxes in anthropic environments in Italy [52,53], obtained data may suggest a potential higher risk of infection in domestic carnivores living in the examined area and frequenting environments frequented also by infected foxes. Interestingly, the two cats found positive in this study lived in the same area as the examined foxes. However, no significant results emerged from statistical analysis in regard to positivity according to the geographical origin of examined domestic carnivores. Nevertheless, it is also possible that the small number of examined and, especially, of positive animals, may have influenced the results of statistical analysis, and further studies are needed to clarify this observation.

## 5. Conclusions

Recurrent cystitis, pollakiuria, and hematuria were the main clinical signs associated with symptomatic urinary capillariosis in pet dogs and cats examined in this study. The detection of only immature eggs or parasite fragments in positive cats may suggest that specific parasitological skills may be required for diagnosing the infection. In light of this, molecular tools could be helpful for future investigations [54].

Obtained data underline the importance of including *Pearsonema* spp. nematodes in the differential diagnosis of dog and cat urinary diseases, especially in animals showing chronic or recurrent cystitis or in those living in environments contaminated by *P. plica* infected foxes.

## Figures and Tables

**Table 1:** Age, sex, lifestyle, presence/absence of clinical signs related to the lower urinary tract, geographical origin (Piedmont, Lombardy, Tuscany), and positivity (n. positive/total examined, %, 95% Confidence Interval) of 26 pet cats and 83 pet dogs examined for *Pearsonema* spp. infection in Italy between May 2015 and March 2017; m: male, f: female.

Patients Description	Cats	Dog
Age range	1.5–14 years old	1–16 years old
Males	14	43
Females	12	40
Mixed breed	19 ^a^	28 ^c^
Pure breed	7 ^b^	55 ^d^
Mainly indoor lifestyle	21	72
Mainly outdoor lifestyle	5	11
Symptomatic	20	62
Asymptomatic	6	21
Piedmont	4 (3 m, 1 f)	10 (6 m, 4 f)
Lombardy	11 (7 m, 4 f)	26 (11 m, 5 f)
Tuscany	11 (4 m, 7 f)	47 (26 m, 21 f)
*Pearsonema* spp. positive	2 (2/26, 7.7%, 95% CI 0–17.9%)	1 (1/83, 1.2%, 95% CI 0–3.6%)

^a^ European; ^b^ 4 Persian, 2 Siamese, 1 Bengal; ^c^ mongrels; ^d^ 9 German shepherd, 7 Boxer, 5 Bernese mountain dog, 4 Yorkshire terrier, 3 Dogo Argentino, 3 Giant poodle, 3 English setter, 2 Pug, 2 Golden retrievers, 2 Jack Russell terrier, 2 Labrador retriever, 2 Pinscher, 2 Cavalier king, 1 American bully, 1 Bernese hound, 1 Bulldog, 1 Pomeranian, 1 Rhodesian ridgeback, 1 Rottweiler, 1 Weimaraner.
